# Scale-up of community action for health: lessons from a realistic evaluation of the *Mitanin* program in Chhattisgarh, India

**DOI:** 10.1186/1753-6561-6-S5-O26

**Published:** 2012-09-28

**Authors:** Devaki Nambiar, Kabir Sheikh, Namrata Verma

**Affiliations:** 1 Governance Hub, Public Health Foundation of India, New Delhi, India

## Introduction

It has long been argued that health for all is most effectively, democratically, and sustainably facilitated through the presence of community health workers at the grassroots. In India, one the few acknowledged successes in community programs include the *Mitanin* program of Chhattisgarh designed and maintained at the scale of the entire state of Chhattisgarh. This program served as prototype for creation of the cadre of an accredited social health activist under India’s flagship program, the National Rural Health Mission. Typical evaluations of scaled-up interventions like this one examine the population-level health outcomes, placing less emphasis on why any such programs succeed or fail. To fill this gap of knowledge, we undertook the realist evaluation of *Mitanin* program. *Mitanin* refers to a female community health worker.

## Methods

We undertook a retrospective realistic evaluation, systematically characterizing the contexts, mechanisms, and outcomes (C-M-O) of community action for health at scale through the *Mitanin* program in Chhattisgarh. Our research drew specifically upon the role of the Chhattisgarh State Health Resource Centre (SHRC) as a technical agency whose genesis and mandate is closely interlinked with the program.

We undertook observations, policy documentary review, in-depth interviews (N=79), and focus group discussions (N=51) with policy and program developers, facilitators and trainers at various levels of the health system, with community health workers, and with representatives of civil society. Four researchers did iterative data analysis following the framework method of qualitative data analysis. Based on an initial free list of themes, interview summaries were coded, and then grouped under meta-themes. Meta-themes were then organized into specific mechanism, context and outcome categories.

## Results

Chhattisgarh’s new statehood in 2000 presented a context of strategic public sector system-building where health and community action were given priority. The creation of SHRC institutionalized a mechanism of plural governance requiring collaboration between a range of public and private stakeholders in funding, selecting, training, and supporting *Mitanins*. This included doctors, bureaucrats, activists, donors, and civil society leaders, combining ‘technical’ or ‘medical-public health’ competencies with ‘socio-political’ empowerment and mobilization. And yet, community action in Chhattisgarh is also shaped by a larger context of tribal alienation and lack of women in statecraft.

A key outcome of efforts by SHRC is the decade-long sustenance of the *Mitanin* program ‘through four Directors, two Chief Ministers and three elections’. While women have not yet occupied leadership positions at SHRC, *Mitanins* are pivotal in grassroots action and are in turn, demanding institutional recognition. SHRC has responded with mechanisms of career building and the creation of additional spaces for local health action with *Mitanin* involvement.

Another mechanism has been ‘recycling’. SHRC functionaries rarely leave the system. Rather, they rotate across various roles at block, district, state and national levels. This retention contrasts against the waning involvement of established non-governmental organizations, and enhances linkages to local rights-based (tribal, food) campaigns.

## Discussion

The SHRC model offers lessons of how large-scale community action in health can be poly-centrically governed. Whereas Chhattisgarh is an exception in that its state health agenda could be formulated afresh, once in place, SHRC mechanisms were intentionally developed at scale requiring both technical and social competencies. Mechanisms were further shaped by the demands of frontline community actors who, over time, gave the programme its unique identity. In the bargain, the engagement of a core group of elite and frontline stakeholders has remained (albeit in different roles and capacities), while relationships with existing community stakeholders in and beyond the health domain have changed.

We therefore recommend that efforts to scale-up community action for health institutionalize broad stakeholdership. Community action efforts require the long-term commitment of leaders to the program, in a framework of plural governance such that no one individual or group can completely change the course of the program; rather interests and ideas can converge and diverge around mechanisms responsive to outcomes at the community level.

We additionally recommend that the *Mitanin* program engages in a more proactive effort to involve marginalized tribal communities in health action, and encourage women’s leadership at the state level.

## Competing interests

Authors declare that they have no conflict of interest.

## Funding statement

The study was funded by the Oxfam India.

